# Social isolation from childhood to mid-adulthood: is there an association with older brain age?

**DOI:** 10.1017/S0033291723001964

**Published:** 2023-12

**Authors:** Roy Lay-Yee, Ahmad R. Hariri, Annchen R. Knodt, Ashleigh Barrett-Young, Timothy Matthews, Barry J. Milne

**Affiliations:** 1 Centre of Methods and Policy Application in the Social Sciences, and School of Social Sciences, Faculty of Arts, University of Auckland, Auckland, New Zealand; 2 Department of Psychology and Neuroscience, Duke University, Durham, NC, USA; 3 Department of Psychology, University of Otago, Dunedin, New Zealand; 4 Department of Social Genetic & Developmental Psychiatry, Institute of Psychiatry, King's College London, London, UK; 5 Department of Statistics, Faculty of Science, University of Auckland, Auckland, New Zealand

**Keywords:** Brain age, cognitive decline, life course, social isolation

## Abstract

**Background:**

Older brain age – as estimated from structural MRI data – is known to be associated with detrimental mental and physical health outcomes in older adults. Social isolation, which has similar detrimental effects on health, may be associated with accelerated brain aging though little is known about how different trajectories of social isolation across the life course moderate this association. We examined the associations between social isolation trajectories from age 5 to age 38 and brain age assessed at age 45.

**Methods:**

We previously created a typology of social isolation based on onset during the life course and persistence into adulthood, using group-based trajectory analysis of longitudinal data from a New Zealand birth cohort. The typology comprises four groups: ‘never-isolated’, ‘adult-only’, ‘child-only’, and persistent ‘child-adult’ isolation. A brain age gap estimate (brainAGE) – the difference between predicted age from structural MRI date and chronological age – was derived at age 45. We undertook analyses of brainAGE with trajectory group as the predictor, adjusting for sex, family socio-economic status, and a range of familial and child-behavioral factors.

**Results:**

Older brain age in mid-adulthood was associated with trajectories of social isolation after adjustment for family and child confounders, particularly for the ‘adult-only’ group compared to the ‘never-isolated’ group.

**Conclusions:**

Although our findings are associational, they indicate that preventing social isolation, particularly in mid-adulthood, may help to avert accelerated brain aging associated with negative health outcomes later in life.

## Background

Humans need connection and interaction with one another to sustain good health (Berkman, Glass, Brissette, & Seeman, 2000; Umberson & Montez, 2010). A negative indicator of social connection is the degree to which an individual is isolated, i.e. lacking contact with others and being alone most of the time (Cacioppo, Hawkley, Norman, & Berntson, 2011; de Jong Gierveld, van Tilburg, & Dykstra, 2006). Social isolation is increasingly recognized as a threat to public health and well-being that requires intervention at a societal level (Holt-Lunstad, Robles, & Sbarra, 2017; Leigh-Hunt et al., 2017). This threat has been further highlighted by the impact on mental health of social restrictions deployed to counter the worst of the COVID-19 pandemic (Aleman & Sommer, 2022; Bu, Steptoe, & Fancourt, 2022; Sommerlad et al., 2022).

But what are the features of social isolation and how might it affect health? The occurrence of social isolation is particularly related to social disadvantage (Rohr et al., 2021) and thus to social exclusion (Holt-Lunstad & Steptoe, 2022; Umberson & Donnelly, 2023). Social isolation can affect individuals at any age, may occur earlier or later during the life course, and may be transient or persistent. Longitudinal investigations of social isolation from childhood to adulthood (Caspi, Harrington, Moffitt, Milne, & Poulton, 2006) are important to understand the development of social isolation and its time-dependent effects. The insights gained from a life-course perspective (Ben-Shlomo & Kuh, 2002) can then be used to identify earlier influences and indicate possible points for intervention to reduce risk of harm in adulthood (Osborn, Weatherburn, & French, 2021). In children, poor family environment has been associated with social isolation (Repetti, Taylor, & Seeman, 2002). This is a barrier to successful attachment with parents or care-givers which impacts on typical child development with ongoing consequences (Bowlby, 1969). Social isolation has been shown to have negative consequences for children's social and emotional functioning (Bukowski & Adams, 2005; Coplan, Ooi, Xiao, & Rose-Krasnor, 2018; Marryat, Thompson, Minnis, & Wilson, 2014). Further, children who have been socially isolated may carry wide-ranging adverse health effects into adulthood, resulting in worse morbidity and mortality [e.g. cardiovascular disease (Caspi et al., 2006), depression (Danese et al., 2009), inflammation (Lacey, Kumaria, & Bartley, 2014), suicide (Rojas, 2018), and premature death (Holt-Lunstad, Smith, Baker, Harris, & Stephenson, 2015)]. Examining trajectories of social isolation from childhood to adulthood may give clues to disentangling the direction of causality and thus enable the potential prevention of poorer health outcomes eventuating from social isolation and its antecedents.

Recent systematic reviews have concluded that positive social connections are associated with slower cognitive decline (Samtani et al., 2022), and that, conversely, a lack of social contact is associated with reduced cognitive function (Kelly et al., 2017), with social isolation specifically related to progressive cognitive decline in older adults (Evans, Martyr, Collins, Brayne, & Clare, 2019). There is also evidence that the positive association between social isolation and memory decline is uni-directional rather than reciprocal, i.e. a higher level of social isolation leads to increased memory decline (Read, Comas-Herrera, & Grundy, 2020). Supporting these linkages, pathological findings show associations between cognitive decline and lower social engagement in healthy older people (Biddle et al., 2019).

While the mechanisms underlying these associations between social integration and health have not been fully determined, behavioral, psychological, and physiological pathways likely play a role (Berkman et al., 2000). For example, being part of a social network tends to promote positive health behaviors, enables access to support and other resources, buffers against stress, and provides a sense of well-being and meaning (Di Marco et al., 2014; Umberson, Crosnoe, & Reczek, 2010). Being actively engaged in social relations plays a foundational role in developing brain reserve (tolerance of the brain's structure to damage) and cognitive reserve (resilience of the brain's function to structural deficits), which act as protective factors against cognitive decline (Barnett, Salmond, Jones, & Sahakian, 2006). The brain's plasticity enables its re-modeling and adaptation in response to internal or external stimuli (Jellinger & Attems, 2013).

Neurophysiological mechanisms – including measures of brain health – have also been implicated in the relationship between social ties and health (Eisenberger & Cole, 2012). The relationship between social isolation and cognitive decline may be mediated by brain age, a measure of brain health. Greater risk of cognitive decline is indicated by deleterious neurochemical changes in the brain that may accompany the aging process (Cleeland, Pipingas, Scholey, & White, 2019). Both structural and functional magnetic resonance imaging (MRI) results have been found to mirror age-related changes in neurochemistry (Eavani et al., 2018). MRI measures of brain structure can be extracted and analyzed to estimate biological brain age (Franke & Gaser, 2019; MacDonald & Pike, 2021). The difference between estimated brain age and actual chronological age [i.e. brain age gap estimate (brainAGE)] can then indicate whether an individual's brain has aged more or less than their peers, calibrated against a population benchmark, reflecting overall brain health and the degree of any underlying neuroanatomical abnormalities present (Liem et al., 2017; Smith, Vidaurre, Alfaro-Almagro, Nichols, & Miller, 2019). Brain age has been shown to predict accelerated cognitive decline (Franke & Gaser, 2012). In particular, older brain age at midlife has been associated with cognitive decline from childhood (Elliott et al., 2021) as well as cognitive impairment in later life (Zheng et al., 2022).

Social isolation has been suggested as a predictor of brain age, e.g. socially isolated individuals showed older estimated brain age relative to controls in a large population-based study (de Lange et al., 2021). However, little is known whether the timing (i.e. life stage of onset) or duration (i.e. persistence across life stages) of social isolation have an impact on brain age. For instance, it is not known whether (i) child-onset social isolation has long-term negative effects on adult brain structure and thus on cognitive function, (2) more proximal (i.e. adult) experiences of social isolation impact cognitive decline, or (iii) prolonged exposure to social isolation has cumulative effects on health (e.g. Caspi et al., 2006).

To understand the impact of timing and duration on cognitive function, this study investigates whether trajectories of social isolation from childhood to midlife are related to estimated brain age gap, a known biomarker of cognitive decline. If so, then the relationship between social isolation and the deterioration of cognitive function may – at least in part – be mediated by deleterious changes in brain structure that accelerate brain aging. We have recently identified four distinct trajectory groups of social isolation using longitudinal data (never-isolated, child-only, adult-only, or child-adult), and have demonstrated that these have different risk factor profiles by family and child characteristics (Lay-Yee et al., 2021). Here, we aim to investigate the degree to which these trajectory groups across a long stretch of the lifespan (from childhood to age 38) are related to brain age in mid-adulthood (assessed at chronological age 45). Specifically, we investigate two research questions:

1. How does the course of social isolation – i.e. membership of trajectory groups: ‘never-isolated’, ‘child-only’, ‘adult-only’, or persistent ‘child-adult’ isolation – impact brain age (at age 45)? We hypothesize that patterns of social isolation in children and adults affect adult brain age, that the adverse effect of social isolation is greater the earlier it occurs during the life course (Vidal-Pineiro et al., 2021), and that any adverse effects accumulate, i.e. there is an effect of persistence (Rudenstine & Galea, 2015). We expect that adult brain age will be older in the isolated groups compared to the ‘never-isolated’ group, greater in ‘child-only’ *v.* ‘adult-only’ groups, and greatest in the ‘child-adult’ group.
2. Can the association between social isolation and brain age, uncovered in (1), be explained by risk factors associated with social isolation also being associated with brain age? We hypothesize that the association between social isolation and brain age will still hold after controlling for factors known to be associated with social isolation.

## Methods

### Data source

We used data from the Dunedin Multidisciplinary Health and Development Study (DMHDS), an ongoing longitudinal investigation of the health and behavior of a complete birth cohort of consecutive births (*N* = 1037) over a one-year period from 1 April 1972 to 31 March 1973 in Dunedin, New Zealand (Poulton, Guiney, Ramrakha, & Moffitt, 2022; Poulton, Moffitt, & Silva, 2015). The DMHDS has been approved by the New Zealand Health and Disability Ethics Committee, and informed consent was obtained from participants. To date, assessments have been carried out at ages 3, 5, 7, 9, 11, 13, 15, 18, 21, 26, 32, 38, and 45. We focus on assessments of outcome at age 45 in 2017–2019 when 94% (*n* = 938) of living cohort members participated in the study. The study sample (*n* = 855) includes participants who had data collected on social isolation at ages 5–11 and 26–38, as well as on brain age in mid-adulthood (at age 45).

### Description of variables

#### Social isolation

*Social isolation* in childhood at ages 5, 7, 9, and 11, and again in adulthood at ages 26, 32, and 38, has been used to statistically derive four trajectory groups: never-isolated (neither isolated as a child nor as an adult); child-only (isolated as a child but not as an adult), adult-only (first became isolated as an adult), and persistent child-adult isolation (isolated both as a child and as an adult) (Lay-Yee et al., 2021; Nagin, 2005). Membership of these groups represents 30-year trajectories in change of social isolation status.

*Child isolation* was assessed by a collection of measures from ages 5 to 11 (Caspi et al., 2006; Danese et al., 2009). When a Study member was 5, 7, 9, and 11 years old, their parent and teacher completed the Rutter Child Scale (Elander & Rutter, 1996), reporting on two items that measure peer problems: ‘tends to do things on his/her own; is rather solitary’ and ‘not much liked by other children’. At each age, scores on these two scale items (0 = doesn't apply, 1 = applies somewhat, 2 = certainly applies) were averaged across the two reporting sources (i.e. parent and teacher).

*Adult isolation* was assessed using *informant report* at ages 26, 32, and 38, whereby up to three informants whom the Study member nominated as ‘knowing them well’ was mailed a questionnaire. At each age, over 90% of Study members had reports from at least two informants, and over 60% had reports from all three informants. At each age, scores on the item ‘seems lonely’ (0 = not a problem, 1 = bit of a problem, 2 = yes, a problem) were averaged across informants.

#### Brain age

The brain age gap estimate (brainAGE) was derived from multiple measures of brain structure – i.e. cortical thickness, surface area, and volumes of subcortical gray matter, white matter, and cerebrospinal fluid – taken from MRI, based on an algorithm developed by Liem et al. (2017). The specific method has been described in detail elsewhere (Elliott et al., 2021). Study members were scanned using a MAGNETOM Skyra 3 T scanner (Siemens Healthcare, Erlangen, Germany) between August 2016 and April 2019. High resolution T1-weighted images, three-dimensional fluid-attenuated inversion recovery images, and a gradient echo field map were obtained. Structural MRI data were analyzed using the Human Connectome Project (HCP) minimal preprocessing pipeline (Glasser et al., 2013). BrainAGE was quantified as the difference between estimated brain age and exact chronological age (birth to MRI scan date).

#### Confounders (childhood)

A complex interplay of factors may be involved in the relationship between social isolation and brain age (de Lange et al., 2021; Richmond-Rakerd et al., 2021). We have found a number of familial and child-behavioral covariates to be associated with social isolation trajectory group membership (Lay-Yee et al., 2021). We adjusted multivariable analyses for these potential confounders of the association between social isolation and brain age. We also adjusted these analyses for socio-demographic factors identified as potentially important from the literature: sex (Umberson, Lin, & Cha, 2022) and family socio-economic status (Schneider, Richard, Younger, & Freeman, 2000).

#### Socio-demographic factors

- *Sex* was defined as binary at birth: male/female.
- Family socio-economic status was measured using the Elley-Irving scale of occupational status (Elley & Irving, 1972), estimated as the average of the higher level of either parent assessed at birth and at ages 3, 5, 7, 9, 11, 13, and 15 years, with scores grouped into low, medium, and high categories.

#### Family factors

- A *teen-aged mother* was defined as being aged 18 or under and coded in a binary variable as ‘yes’ or ‘no’.
- We assessed whether the child had a *single parent* for at least one year up to age 11 as a binary variable coded ‘yes’ or ‘no’.
- We measured c*hange in residence* as the number of times that this occurred up to age 11.
- An index of *maltreatment* was formed by combining measures of atypical maternal behavior (age 3), harsh discipline (ages 7 and 9), disruptive caregiver changes from birth to age 11, exposure to physical abuse from birth to age 11, and exposure to sexual abuse from birth to age 11 (Caspi et al., 2002).

#### Child-behavioral factors

- *Self-control*, the ability to regulate emotions and behavior, was assessed using a scale combining measures of lack of control (ages 3 and 5), impulsivity (ages 5, 7, 9, and 11), and hyperactivity (ages 5, 7, 9, and 11) (Moffitt et al., 2011). The scale was split into quintiles for the purposes of analysis (quintile 1 = highest self-control; quintile 5 = lowest self-control).
- When a Study member was 5, 7, 9, and 11 years old, their parent and teacher completed the Rutter Behavior Scales (Elander & Rutter, 1996). Scores on the *worried/fearful* scale, a measure of childhood internalizing symptoms, were averaged across rater and age and split into quintiles for analysis and interpretation (note that items relating to social isolation were not used in the construction of the worry/fearful scale).

### Data analysis

We previously derived a typology comprising four groups according to the onset and duration of social isolation: never-isolated (*n* = 710, 71.6% of the cohort), child-only (*n* = 142, 14.3%), adult-only (*n* = 100, 10.1%), and child-adult (*n* = 40, 4.0%) (Lay-Yee et al., 2021). Here, we employed that same typology of trajectory groups to examine its relationship to adult brain age (at chronological age 45).

Firstly, we ascertained the mean brainAGE (with standard deviations) for each trajectory group and across categories within potential confounders, and then tested to see if there were bivariate associations (*p* < 0.05). Secondly, we used linear regression analysis to ascertain whether brainAGE was predicted by different trajectory group membership, while controlling for childhood factors previously shown to be important risk factors for social isolation, i.e. having a teenaged mother, having a single parent, changes in residence, maltreatment, self-control, and worry/fearfulness (Lay-Yee et al., 2021); as well as sex and family socio-economic status. We report model-adjusted marginal means (with standard errors) of brainAGE by trajectory group and confounders, and beta coefficients (with 95% confidence intervals and *p* values) to indicate the importance of trajectory group. To check that the differences in trajectory group size were not affecting the results of our regression analysis, we carried out online Supplementary analysis based on the average scores across the four measures in childhood and the three measures in adulthood, converted to *z* scores for ease of interpretation. Analyses were carried out using Stata 16.0 (StataCorp, 2019).

## Results

### Bivariate associations

Table 1 shows brainAGE stratified by the trajectory groups, indicating very strong associations though without any covariate adjustment. There are clear differences between group means, particularly between the ‘adult only’ (mean 1.78, s.d. 7.71, *p* = 0.012) and persistent ‘child-adult’ (mean 3.47, s.d. 8.85, *p* = 0.006) groups and the ‘never-isolated’ group (mean −0.58, s.d. 7.91). Results suggest that there is a sizeable 4-year difference between brainAGE for the persistent ‘child-adult’ group and the ‘never-isolated’ group, using our trajectory classification. Family circumstances in childhood showing an association with brainAGE were socio-economic status, change in residence, and maltreatment. Sex was also significantly related to brainAGE though child behavioral factors were not.

**Table 1.** Brain age gap estimate (brainAGE) at age 45 years by ‘trajectory’ group and family-child factors

Covariates	BrainAGE at 45			
	*n*	Mean^a^	Standard deviation	*p*^b^
Total sample	855	−0.05	8.00	–
Trajectory group				
Never-isolated	623	−0.58	7.95	Ref
Child-only	121	0.62	7.89	0.127
Adult-only	80	1.78	8.09	0.012*
Child-adult	31	3.47	7.62	0.006*
Sex				
Female	425	1.03	7.96	Ref
Male	430	−1.11	7.89	<0.001*
Socio-economic status				
Low	169	2.08	9.04	Ref
Medium	545	−0.52	7.67	<0.001*
High	137	−1.04	7.25	0.001*
Teen-aged mother				
No	764	−0.13	7.98	Ref
Yes	88	0.60	8.16	0.416
Single parent for at least a year, up to age 11				
No	728	−0.25	7.91	Ref
Yes	116	1.30	8.68	0.054
Number of changes in residence				
0	249	−0.98	7.45	Ref
1	193	1.10	7.88	0.006*
2	140	−0.24	7.56	0.379
3+	273	0.10	8.67	0.120
Maltreatment				
None	549	−1.12	7.63	Ref
Probable/severe	306	1.89	8.27	<0.001*
Self-control				
Quintile 1 (high)	166	−0.35	8.00	Ref
Quintile 2	182	−0.86	7.49	0.555
Quintile 3	174	−0.55	7.42	0.822
Quintile 4	176	0.49	8.73	0.332
Quintile 5 (low)	157	1.18	8.22	0.085
Worry/fearfulness				
Quintile 1 (low)	117	−0.44	6.89	Ref
Quintile 2	191	−1.28	7.77	0.368
Quintile 3	169	−0.33	7.65	0.909
Quintile 4	196	0.69	8.34	0.224
Quintile 5 (high)	182	0.98	8.66	0.133

* *p* < 0.05.

a Positive values indicate older and negative values younger estimated brain age compared with chronological age.

b One-way analysis of variance of brain age gap by trajectory group and covariates respectively.

### Covariate-adjusted associations

Table 2 shows that social isolation retained an effect (now attenuated) on brainAGE independent of early family and child factors, particularly in the case of ‘adult-only’ isolation (marginal mean 1.73, s.e. 0.89, *p* = 0.024). It should be noted that persistent ‘child-adult’ isolation had the highest mean brainAGE (2.29 years), but the difference between the persistent ‘child-adult’ and ‘never-isolated’ groups was not statistically significant.

**Table 2.** Relationship between ‘trajectory’ group and brain age gap estimate (brainAGE) at age 45 years

Trajectory group	brainAGE at 45		
	Marginal mean (s.e.)^a^	*β* (95% CL)	*p*^b^
Never-isolated	−0.43 (0.32)	Ref	Ref
Child-only	−0.09 (0.74)	0.34 (−1.28, 1.96)	0.681
Adult-only	1.73 (0.89)	2.16 (0.29, 4.03)	0.024*
Child-adult	2.29 (1.53)	2.72 (−0.36, 5.81)	0.084
Control variables			
Sex (*Female* *=* *Ref*)			
Male		−2.32 (−3.44, −1.20)	<0.001*
Socio-economic status (*Low* *=* *Ref*)			
Medium		−1.95 (−3.35, −0.54)	0.007*
High		−2.35 (−4.21, −0.49)	0.013*
Teen-aged mother (*No* *=* *Ref*)			
Yes		−0.35 (−2.20, 1.51)	0.713
Single parent for at least a year, up to age 11 (*No* *=* *Ref*)			
Yes		−0.25 (−1.88, 1.39)	0.767
Change in residence (*0* *=* *Ref*)			
1		2.10 (0.61, 3.58)	0.006*
2		0.37 (−1.29, 2.03)	0.663
3+		0.15 (−1.29, 1.59)	0.840
Maltreatment (None = Ref)			
Probable/severe		2.49 (1.30, 3.67)	<0.001*
Self-control (*Quintile 1* (*high*) *=* *Ref*)			
*Quintile 2*		−0.70 (−2.37, 0.97)	0.411
*Quintile 3*		−1.10 (−2.83, 0.63)	0.211
*Quintile 4*		0.58 (−1.20, 2.36)	0.524
*Quintile 5* (*low*)		0.31 (−1.62, 2.24)	0.753
Worry/fearfulness (*Quintile 1* (*low*) *=* *Ref*)			
Quintile 2		−1.46 (−3.28, 0.36)	0.116
Quintile 3		−1.20 (−3.10, 0.69)	0.213
Quintile 4		−0.52 (−2.39, 1.36)	0.589
Quintile 5 (high)		−0.26 (−2.24, 1.71)	0.793
Model *R*^2^		0.0918	

* *p* < 0.05.

a Positive values indicate older and negative values younger estimated brain age compared with chronological age.

b Multi-way analysis of variance model, controlled for sex, socio-economic status, teenaged mother, single parent, change in residence, maltreatment, self-control, and worry/fearfulness.

### Supplementary analysis

Differences in trajectory group size did not affect the results of our regression analysis. Re-analysis using continuous isolation scores (rather than groups) showed that adult isolation was important and child isolation was not, corroborating our original analysis of trajectory groups. There was no interaction indicating that there was no extra effect of having high scores on both adult and child isolation (see online Supplementary Table S1).

## Conclusions

This paper presents the findings of a longitudinal study of social isolation from a New Zealand population-representative birth cohort followed from birth to mid-adulthood. We used four trajectory groups, identified in an earlier study (Lay-Yee et al., 2021), that mapped well to the onset and persistence of social isolation: never-isolated, child-only, adult-only, and child-adult. The trajectory groups were shown not to differ by sex, while the child-adult group was found to consistently have the worst profile across both family-environment and child-behavior factors. Our research questions aimed to test if membership of these trajectory groups was associated with older brain age at age 45, and, if so, whether any association was upheld after controlling for relevant family and child confounders.

### Principal findings

Our findings corroborate and add to the evidence that there is a positive association between adult social isolation and brain age (e.g. de Lange et al., 2021). We show, in analyses adjusted for confounders, that individuals with adult-only social isolation had mean estimated brain age that was 1.73 years older than individuals who never experienced social isolation. The pattern of relationship between social isolation trajectory groups and brain age suggested in unadjusted analysis disappeared in the presence of confounders. The mean estimated brain age of the persistently isolated (child-adult) group differed from that of the never-isolated group in unadjusted analyses (3.47 years older) but was attenuated and non-significant in adjusted analyses (2.29 years older). The mean estimated brain age for the child-only group did not differ from that for the never-isolated group, either in unadjusted or adjusted analyses. In general, it appeared that the experience of social isolation in adulthood – with or without childhood isolation – had the greatest impact on estimated brain age (Matthews et al., 2022).

### Implications

The theoretical significance of this study lies in its focus on the longitudinal effects of social isolation on an adult outcome (de Jong Gierveld et al., 2006), specifically brain age. Our finding suggesting that adult isolation has a greater influence on brain age than persistent (child-adult) isolation requires further investigation, as it seems to run counter to the hypothesis that long-term exposure to social isolation leads to chronic biological embedding, and, in turn, to older brain age. An alternative explanation may be reverse causation – i.e. older brain age leads to social isolation – which could be tested in future with repeated measurements of both social isolation and brain age. Our finding that child-only isolation was not related to older brain age is also unexpected and against our hypothesis that earlier exposure would have a greater effect on outcome. We speculate that the child's brain may be resilient to or has the capacity to recover from being socially isolated, particularly if isolation is not experienced again as an adult. Nevertheless, the current study helps to elucidate the contribution of social isolation to older brain age (while controlling for confounders located in childhood) and adds novel evidence as to its detrimental effect on brain aging. Social isolation, defined as an extreme lack of social ties, is a relational phenomenon that may become biologically embedded in the person who experiences it, i.e. the brain's structure and function are able to be shaped by social relations (Bzdok & Dunbar, 2022; Morese & Palermo, 2022). For example, social isolation reduces access to the stimulation that is required to build stocks of cognitive reserve, thus diminishing the buffer against cognitive decline (Evans et al., 2018). Potential neurophysiological mechanisms driving the association between social isolation and brain health include effects on brain chemistry, oxidative stress, immune response, inflammation, impaired myelination, synapse loss, and allostatic load (Drinkwater, Davies, & Spires-Jones, 2021; Eisenberger & Cole, 2012). At a molecular level, the disruption of gene regulation and expression in the brain may be underpinning these physiological effects of social isolation (Arzate-Mejía, Lottenbach, Schindler, Jawaid, & Mansuy, 2020).

Even if the temporal relationships reported here are not causal, they suggest that social isolation at different life stages – particularly in mid-adulthood – is associated with older brain age. The association is strong enough for even mild levels of cognitive impairment to be linked to social isolation (Ishikawa, Davis, Chen, & Lim, 2022). Brain age, a global measure of brain health, may be mediating the effect of social isolation on cognitive decline, given that both social ties and brain health have been associated with cognitive function. For example, it has been found that gray matter volume, a specific measure of brain health, partly mediates the longitudinal association between social isolation and cognitive function (Shen et al., 2022). Finally, we may speculate that there are implications for understanding dementia, a pathological form of cognitive dysfunction, given that a lack of social contact has been associated with elevated dementia risk (see reviews by: Desai, John, Stott, & Charlesworth, 2020; Kuiper et al., 2015; Penninkilampi, Casey, Singh, & Brodaty, 2018; Ren et al., 2023; and Sommerlad, Ruegger, Singh-Manoux, Lewis, & Livingston, 2018), and that older brain age has been considered to be a biomarker for (Gaser, Franke, Klöppel, Koutsouleris, & Sauer, 2013; Wang et al., 2019) and midlife antecedent of (Reuben et al., 2022) dementia.

In terms of policy and practice, understanding the influence of life-course differences in onset and persistence of social isolation on brain health should inform screening and intervention strategies. The evidence on interventions is currently sparse and the effectiveness of interventions is likely to vary by life stage. Our study suggests that reducing social isolation could be used to improve brain health and subsequently prevent, arrest, or delay cognitive decline. Timely detection of cognitive decline, before the appearance of significant clinical symptoms, is important as interventions will be more likely to be effective earlier in the life course. Further, our findings may inform the specification of interventions to suit sub-populations or individuals at different life stages (early, mid, and later) and in recognizing vulnerable groups (by sex, social disadvantage, or family and childhood factors) that might benefit from public services. In particular, our finding of an adult effect and tentatively a persistent effect of social isolation on brain age may indicate points for intervention. For example, providing early support for vulnerable adults in midlife may be crucial to preventing the onset (and to interrupting the persistence) of social isolation, and, in turn, slowing brain aging and reducing the occurrence of cognitive decline in later adulthood. In fact, a longitudinal intervention study of healthy adults already has reported that mental training improved cognitive and social skills and that this was correlated with MRI-based measures of increased cortical thickness (Valk et al., 2017). Our finding that child-only social isolation did not contribute to older brain age bodes well for intervention strategy: the child brain appears to be robust and it is not too late to intervene in adolescence or adulthood.

### Strengths and limitations

The main strengths of this study are the longitudinal assessment of social isolation from childhood to adulthood using trajectory modeling, and the measurement of brain structure for a large general population sample about which there have been detailed life-course phenotypic information collected. This enables not only the analysis of social isolation onset but also of any persistence across the life course. We were able to use an existing typology of four trajectory groups derived from child and adult measures of social isolation.

Limitations involve, first, that our heuristic social isolation measures were not designed specifically to measure social isolation and only cover one aspect of social isolation, i.e. social disconnectedness (Cornwell & Waite, 2009), and did not assess loneliness, i.e. how the experience was perceived by participants (Perlman & Peplau, 1981). Social isolation and loneliness have been shown to be distinct entities that are not highly correlated and may have independent effects on health outcomes (Perissinotto & Covinsky, 2014; Valtorta, Kanaan, Gilbody, & Hanratty, 2016). A recent review concluded that both social isolation and loneliness affect cognitive health, though their effects may be mediated differently, i.e. by a lack of stimulation (in the former case) and depression (in the latter) (Cardona & Andrés, 2023). Our social isolation measures have been associated with cardiovascular disease (Caspi et al., 2006), various age-related diseases (Danese et al., 2009), and chronic inflammation (Lacey et al., 2014), while our derived trajectory groups have been related to depression and suicide (Lay-Yee et al., 2023), and retinal neuronal thickness (Barrett-Young et al., 2023). Second, we have not accounted for confounding by factors contemporaneous with adulthood. Third, while we accounted for likely confounding factors from childhood, we cannot infer causality and so cannot rule out reciprocal effects, i.e. social isolation is associated with, but may not necessarily directly cause, changes in brain structure, while reverse causation may also operate. Fourth, while the estimated mean brain age gap for the child-adult group was the largest of all trajectory groups, even after controlling for potential confounding variables that were elevated in this group (Lay-Yee et al., 2021), small numbers (*n* = 31 in the child-adult group) may have hampered the detection of an association between persistent social isolation and brain age. Fifthly, we did not have the requisite data to cover adolescence, an important life stage. Finally, the findings derive from analyses of a predominantly ethnically European cohort in New Zealand so may not be generalizable to all populations.

### Future research

This paper examines risk factor levels for groupings by social isolation that have an intrinsic longitudinal dimension. More sophisticated longitudinal analyses with the requisite data offer the possibility of assessing changes in brain age in tandem with changes in social isolation, especially in the transition from childhood to adulthood. Sharp inflection points during the life course – perhaps indicating sensitive or critical periods – could thus be identified where interventions could reasonably be targeted to good effect. Considering later circumstances that might be more relevant to an adult outcome would clarify the effect of social isolation trajectories. Further studies are required to examine the causal mechanism whereby social isolation affects brain health outcomes (Cené et al., 2022) or whether the relationship is stronger in the reverse direction.

### Conclusion

This study shows that social isolation, particularly when occurring in adulthood, is associated with older estimated brain age in mid-adulthood. There are implications for the screening for and treatment of cognitive decline. Points of intervention during the life course are suggested by our findings, addressing the onset and persistence of social isolation to improve brain health, and thereby contributing to the prevention or mitigation of cognitive decline and its private and public burdens.

## Supporting information

Lay-Yee et al. supplementary material Lay-Yee et al. supplementary material

## Supplementary material

The supplementary material for this article can be found at https://doi.org/10.1017/S0033291723001964
