# Supplementary material for: Social isolation from childhood to mid-adulthood: is there an association with older brain age?
Source: Psychol Med. 2023 Jul 24;53(16):7874–82. doi: 10.1017/S0033291723001964 (PMC10755222; doi:10.1017/S0033291723001964)
Supplement: Lay-Yee et al. supplementary material [file S0033291723001964sup001.docx]

**Table S1.** Relationship between isolation scores and brainAGE at age 45 years

|  | **brainAge at 45** | | | | | |
| --- | --- | --- | --- | --- | --- | --- |
| **Social isolation** | β | SE | 95% CL | | t | P>\|t\| ^a^ |
| child | .32 | .28 | -.23 | .87 | 1.14 | 0.254 |
| adult | .88 | .29 | .30 | 1.45 | 2.99 | 0.003 * |
| child*adult interaction | -.17 | .25 | -.66 | .32 | -0.68 | 0.498 |

a – linear regression model controlled for sex, socio-economic status, teenaged mother, single parent, change in residence, maltreatment, self-control, and worry/fearfulness

* p <0 .05
